# Understanding the Predictors of Missing Location Data to Inform Smartphone Study Design: Observational Study

**DOI:** 10.2196/28857

**Published:** 2021-11-16

**Authors:** Anna L Beukenhorst, Jamie C Sergeant, David M Schultz, John McBeth, Belay B Yimer, Will G Dixon

**Affiliations:** 1 Centre for Epidemiology Versus Arthritis Manchester Academic Health Science Centre University of Manchester Manchester United Kingdom; 2 Department of Biostatistics Harvard T. H. Chan School of Public Health Harvard University Boston, MA United States; 3 Centre for Biostatistics University of Manchester Manchester United Kingdom; 4 Centre for Atmospheric Science Department of Earth and Environmental Sciences University of Manchester Manchester United Kingdom; 5 Centre for Crisis Studies and Mitigation University of Manchester Manchester United Kingdom; 6 NIHR Greater Manchester Biomedical Research Centre Manchester Academic Health Science Centre University of Manchester Manchester United Kingdom

**Keywords:** geolocation, global positioning system, smartphones, mobile phone, mobile health, environmental exposures, data analysis, digital epidemiology, missing data, location data, mobile application

## Abstract

**Background:**

Smartphone location data can be used for observational health studies (to determine participant exposure or behavior) or to deliver a location-based health intervention. However, missing location data are more common when using smartphones compared to when using research-grade location trackers. Missing location data can affect study validity and intervention safety.

**Objective:**

The objective of this study was to investigate the distribution of missing location data and its predictors to inform design, analysis, and interpretation of future smartphone (observational and interventional) studies.

**Methods:**

We analyzed hourly smartphone location data collected from 9665 research participants on 488,400 participant days in a national smartphone study investigating the association between weather conditions and chronic pain in the United Kingdom. We used a generalized mixed-effects linear model with logistic regression to identify whether a successfully recorded geolocation was associated with the time of day, participants’ time in study, operating system, time since previous survey completion, participant age, sex, and weather sensitivity.

**Results:**

For most participants, the app collected a median of 2 out of a maximum of 24 locations (1760/9665, 18.2% of participants), no location data (1664/9665, 17.2%), or complete location data (1575/9665, 16.3%). The median locations per day differed by the operating system: participants with an Android phone most often had complete data (a median of 24/24 locations) whereas iPhone users most often had a median of 2 out of 24 locations. The odds of a successfully recorded location for Android phones were 22.91 times higher than those for iPhones (95% CI 19.53-26.87). The odds of a successfully recorded location were lower during weekends (odds ratio [OR] 0.94, 95% CI 0.94-0.95) and nights (OR 0.37, 95% CI 0.37-0.38), if time in study was longer (OR 0.99 per additional day in study, 95% CI 0.99-1.00), and if a participant had not used the app recently (OR 0.96 per additional day since last survey entry, 95% CI 0.96-0.96). Participant age and sex did not predict missing location data.

**Conclusions:**

The predictors of missing location data reported in our study could inform app settings and user instructions for future smartphone (observational and interventional) studies. These predictors have implications for analysis methods to deal with missing location data, such as imputation of missing values or case-only analysis. Health studies using smartphones for data collection should assess context-specific consequences of high missing data, especially among iPhone users, during the night and for disengaged participants.

## Introduction

Smartphones offer opportunities to collect sensor data frequently from people’s daily lives and to determine their exposures or behaviors. Smartphone location data can be collected frequently (eg, daily, hourly, continuously) over sustained periods of time [1]. Studies have used these data to quantify exposure to weather [2,3], air pollution [4], vicinity to tobacco outlets [5], or to deliver context-aware messages when participants visited health facilities [6,7]. Smartphones can provide complete and accurate location data, especially when participants are provided with study smartphones, studies are short, and data are collected nearly continuously [8,9]. However, in large-scale epidemiological studies, location data are often collected for longer periods, less frequently, and from participants’ own smartphones. In these cases, missing data are more common than when using research-grade location trackers [4,10,11]. In observational research studies, missing data can result in the loss of power, selection bias, and misclassification of participants’ exposure or behavior [12]. In trials, it could hamper safe and effective delivery of context-aware interventions that rely on location data [13].

To anticipate the potential impact of missing location data on study findings, we need to better understand how often, when, and why location data are missing. Previous smartphone studies have reported the amount of missing location data [4,10,14,15]. However, they typically did not investigate differences in missing data over time [4,10,14,15], between participants [4,10,14,15], or between operating systems [4,14]. In addition, they have limitations of small sample sizes.

We therefore investigated the distribution of missing location data over time, predictors of missing location data, and between-participant differences. We used data from a longitudinal smartphone study with 9665 participants using Android phones or iPhones. We anticipate that understanding the predictors of missing location data could inform researchers who want to improve data completeness during study design and data collection.

## Methods

### Ethics Approval and Consent to Participate

The University of Manchester Research Ethics Committee (reference, ethics/15522) and the National Health Service Integrated Research Application System (reference 23/NW/0716) approved this study. Participants were required to provide electronic consent for study inclusion. Further details are available elsewhere [2,3].

### Study Design

We performed a secondary analysis of the data from an observational smartphone study that analyzed the association between weather conditions and chronic pain in the United Kingdom (study name: *Cloudy with a Chance of Pain*) [3]*.* In this study, we collected self-reported pain levels from a large group of people with chronic pain such as osteoarthritis, rheumatoid arthritis, or migraine. The exposure of interest was daily average weather conditions (ie, temperature, relative humidity, wind speed, and air pressure). To determine what daily average weather conditions participants were exposed to, the app recorded participants’ geolocation, which we could link to weather reports from local weather stations. The analysis of the weather and pain association and the details of data collection are described elsewhere [2,3].

### Data Collection

People with chronic pain downloaded the app onto their Android phones or iPhones, provided informed consent, and reported baseline participant characteristics (eg, sex, year of birth, self-reported weather sensitivity). At local time of 6:24 PM each day, participants received a push notification to complete a survey, rating 10 aspects of symptoms, behavior, and well-being. To obtain weather data from the closest weather station, geolocation was required. The app was programmed to record geolocation each hour on the hour; thus, the app would ideally obtain 24 geolocations each day. The app used GPS (outdoors) and network signals (inside buildings) to determine the latitude and longitude. The app’s ability to record geolocations depended on (1) the participant granting the app access to their geolocation and (2) the participant switching on the location services on their phone. Upon downloading the study app, the participants were requested access to their geolocation. Access to geolocation was voluntary; participants who provided the app with access to their geolocation could retract access at any time or switch off location services temporarily or permanently, in which case the app would not be able to record the participant’s location. The app recorded the operating system of the smartphone, but this feature was introduced 1 week after the recruitment launch and was not collected for early enrollers.

### Data Preparation and Eligible Participants

We investigated location-data completeness on calendar days that a participant completed the survey. Participants were eligible if they completed the survey at least once, excluding the day of enrollment. This exclusion ensured comparability of participant days, as recording 24 geolocations would be unlikely on the day of download. For each participant, we selected all days with survey data. For each full clock hour, we added indicators for (1) location data (1 if observed, 0 if missing), (2) number of days since the most recent survey completion (0 if less than 24 hours ago, 1 if 24-47 hours ago, etc), (3) time in study (days since first survey submission), and (4) time (weekday or weekend, part of the day where night was considered as midnight to 5:59 AM, morning as 6 AM to 11:59 AM, afternoon as noon to 5:59 PM, evening as 6 PM to 23:59 PM, and hour of the day). In addition, we added indicators for variables that did not change over time: (1) participant characteristics (eg, sex, age, self-reported weather sensitivity) and (2) operating system (eg, iPhone operating system, Android, or unknown).

### Data Analysis

We reported the number of eligible participants and their characteristics. We reported location-data completeness (1) per day (number of recorded locations during a day), (2) per hour for each clock hour (percentage of participant days with a recorded location data at that hour), (3) per hour for the 4 hours before and after survey completion, and (4) averages per participant (median number of recorded locations) for all participants and stratified by operating system. We investigated predictors of the outcome “presence of a location data point” (0 if missing, 1 if observed for a given full clock hour) with a logistic regression model with a participant-specific random intercept for within-participant correlation between repeated measurements [16-18]. A multivariable model identified whether the likelihood of the missing location data were associated with time indicators (ie, weekdays vs weekend days, part of the day), participant characteristics (ie, age, sex, self-reported weather sensitivity dichotomized around the median), operating system on their phone, survey compliance (ie, days since previous survey entry), or time in study (ie, days since first survey entry). Only participants with complete data for all covariates contributed information to the model. We estimated 95% CIs with 1000 simulations as recommended in [19]. Models were fitted in R (R Core Team) version 3.6 with the package lme4 [18]; odds ratios (ORs) and CIs were estimated using the merTools package [20].

## Results

**Figure 1:**
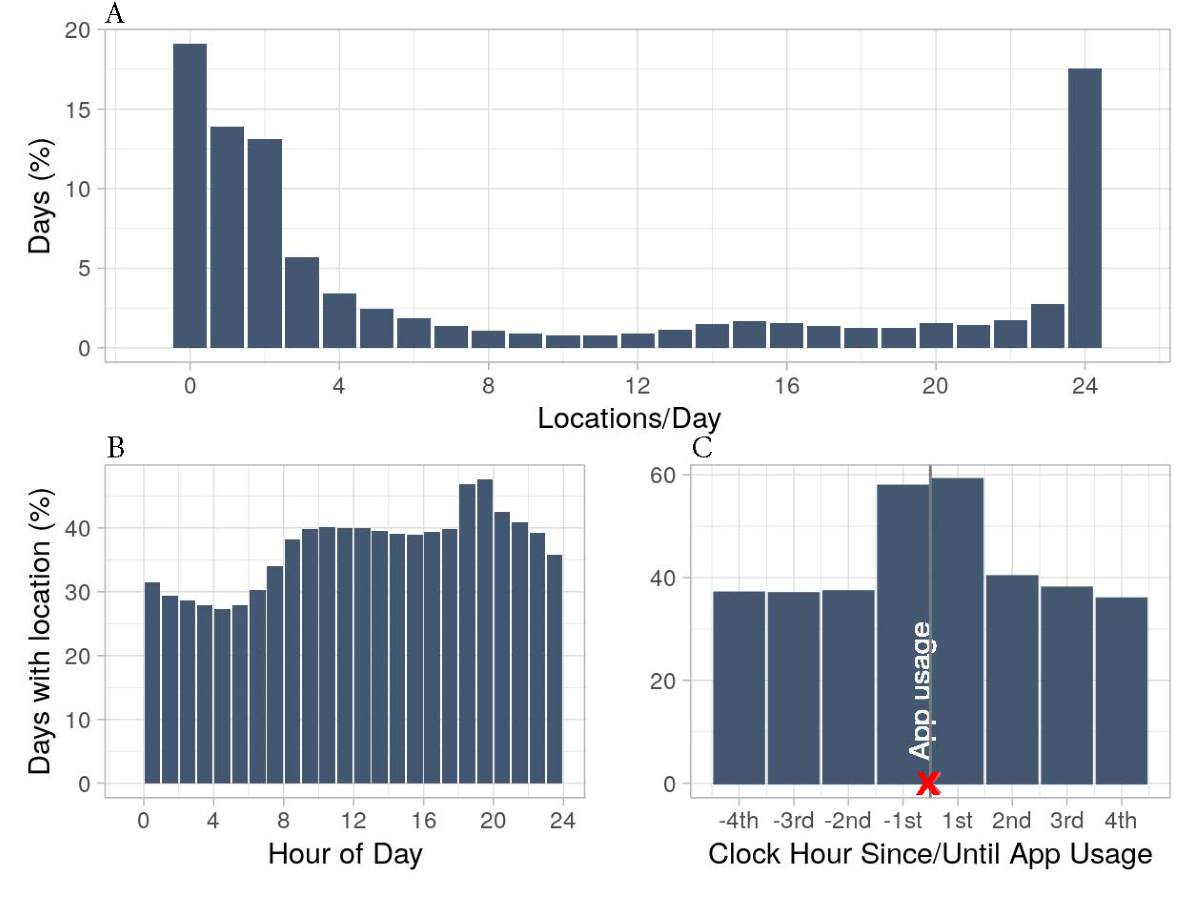
Data completeness. A: Distribution of participant days with a recorded location, stratified per hour of the day (N=488,400). B: Data completeness per hour of the day (N=24 x 488,400). C: Data completeness around the moment of survey completion (N=24 x 488,400). The red X marks app usage, and 1st is the first full clock hour after data entry.

The app was downloaded by 13,207 participants, of which 9665 were eligible for inclusion (mean age 49 [SD 13] years; females, 7211/9665, 74.6%). These participants contributed to 488,400 participant days (median 14 eligible days/participant; IQR 4-60 days/participant). Of 9665 participants, 3109 (32.2%) used an Android phone, 1930 (19.9%) an iPhone, and the operating system was unknown for the remaining 4626 (47.6%) participants. We expected 11.72 million location data points or clock hours: 24 for each hour in the 488,400 participant days. Of 11.72 million hours, the app collected only 4.36 million clock hours (37.2%), resulting in missing data for the remaining 7.36 million clock hours. Data completeness per participant day varied from no location data (0/24) to fully complete data (24/24, Figure 1A, median 3, IQR 1-19). Location data were complete (24/24) for 17.5% (85,606/488,400) of participant days. Participant days with no location (93,255/488,400, 19.1%), 1 location (67,963/488,400, 13.9%), or 2 locations (64,207/488,400, 13.1%) were also common. Location was most often recorded at 7 PM (232,295/488,400, 47.5% of participant days; Figure 1B) just after the default notification of 6:24 PM. Locations were least often recorded between midnight and 6 AM. Location data were often recorded for the hour before survey completion (281,767/487,391, 57.8%) and the hour after survey completion (257,743/436,263, 59.1%; Figure 1C).

**Figure 2:**
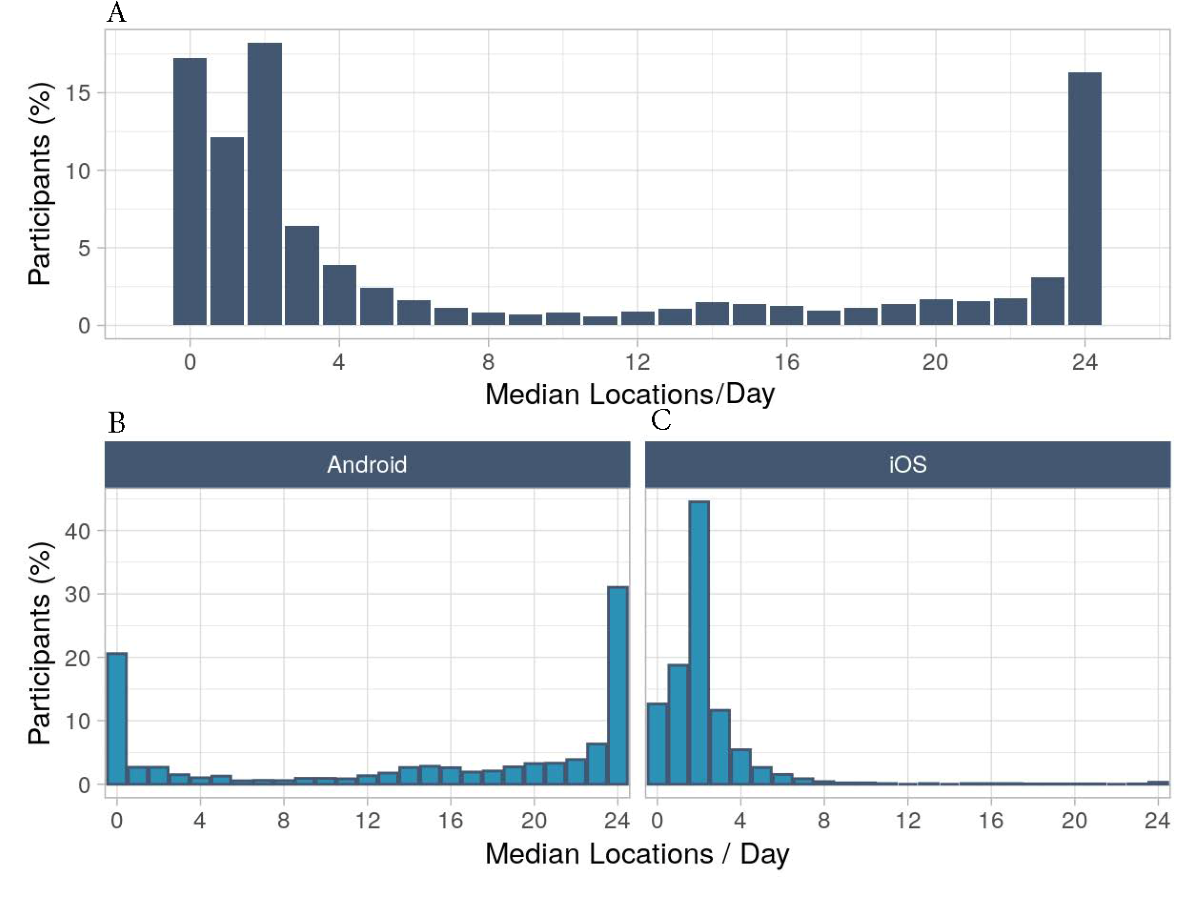
Median locations per day per participant. A: All participants (N=9665). B: Stratified by operating system for 3109 Android users and 1930 iPhone users. iOS: iPhone operating system.

For most participants, the app collected a median of 2 out of a maximum of 24 locations (1760/9665, 18.2% of participants), no location data (1664/9665, 17.2%), or complete location data (1575/9665, 16.3%; Figure 2A). Stratification by phone operating system and participant characteristics showed that 31.0% (965/3109) of Android users had 24 recorded locations on average versus less than 1% (6/1930) of iPhone users (Figure 2B). Android users usually had averages of 24 (out of 24) (965/3109, 31.0%) or 0 (out of 24) locations (640/3109, 20.6%). iPhone users usually had averages of 2 (out of 24) (859/1930, 44.5%) or 1 (out of 24) (362/1930, 18.8%) location, while only 0.03% (6/1930) had averages of 24 (out of 24) locations.

The generalized linear mixed-effects model estimated whether time indicators, operating system, time since previous survey completion, or participant characteristics predicted the presence of a location data point (N=4435). The presence of a location data point was strongly predicted by the operating system and the part of the day (Table 1). The odds of a recorded location were the highest for Android phones (OR 21.91, 95% CI 19.53-26.87, referent: iPhone operating system) and during the afternoon (OR 1.18, 95% CI 1.18-1.20, referent: morning). The odds of a recorded location were lower in the weekends (OR 0.94, 95% CI 0.94-0.95, referent: weekdays) and if previous survey completion was longer ago (OR 0.95 per additional day, 95% CI 0.95-0.95) and marginally lower if a participant’s time in study was longer (OR 0.998, 95% CI 0.9984-0.9985). Participant characteristics (eg, age, sex, self-reported weather sensitivity) did not predict the probability of location data.

**Table 1:** Results of the generalized linear mixed-effects model estimating the odds of having a recorded location (N=4435).

Variable	category	Odds ratio (95% CI)
**Day of the week**		
	Weekdays	Referent
	Weekend	0.94 (0.94-0.95)
**Part of the day**		
	Morning	Referent
	Afternoon	1.19 (1.18-1.20)
	Evening	1.11 (1.10-1.11)
	Night	0.37 (0.37-0.38)
Time in study	Per day	0.99 (0.99-1.00)
**Operating system**		
	iPhone operating system	Referent
	Android	22.91 (19.53-26.87)
Time since previous survey completion	Per day	0.96 (0.96-0.96)
Age	Per 10 years	1.00 (1.00-1.01)
**Sex**		
	Female	Referent
	Male	0.99 (0.80-1.22)
**Weather sensitivity**		
	Weak	Referent
	Strong	0.98 (0.84-1.15)

## Discussion

### Principal Findings

In our study, location data collected from participants’ smartphones were missing for 63% of the intended hours (7.36 million/11.72 million). This percentage is higher than that reported in 5 other studies, reporting 26% [4], 28% [14], and 50% [10,15,21] of missing data. This difference may be due to the choices during the analysis: 3 studies excluded participants with the highest amounts of missing data and only investigated Android users, possibly resulting in an underestimation of the overall percentage of missing location data [4,14,21]. The other 2 studies sampled location continuously multiple times per hour for a few minutes, suggesting that our findings may not generalize to higher frequencies of location data collection [10,15].

### Why Do Time Indicators and Operating System Predict Location-Data Completeness?

Missing data were predicted by part of the day, time since previous survey completion, and participants’ operating system. Missing data at night might be caused by people being indoors where GPS signals are unavailable [11] or by their phones being switched off in airplane mode or out of battery [11,22]. Location data were most complete in the hour before and after survey completion, showing that apps are more likely to record the last known location upon restarting the app and the location on the clock hour after. In addition, we found a small but significant reduction in odds of a recorded location over time. Lower location-data completeness when people stay longer in a study is in line with the findings reported previously [22]. Less than 1% of iPhone users had complete location data. Other studies of smartphone data corroborate our finding of higher missing sensor data in iPhone users compared to Android users. iPhone’s operating system refuses geolocation requests by apps more often compared to Android. Reasons for refusing geolocation requests are, for example, to reduce the phone’s power consumption or to prioritize sensor data collection by other apps [10,15,23,24]. Of note, some studies have succeeded in obtaining higher coverage location data from iPhones compared to Android phones in spite of these operating system–specific differences [22,25]. This finding suggests that the research app used to collect data and the way this app interacts with the operating system may influence the amount of missing data. Experimental studies could further investigate this, as we cannot exclude the role of other differences between this study and our own study, such as the investigated population (eg, mean age 48 years in our study, but mean age 25 years in [22]) and sampling frequency (once an hour in our study; continuously for 1 minute every 10 minutes in [15,22]).

### Implications: Consequences of Missing Data Are Context-Specific

Although missing location data reduce precision, they do not necessarily reduce a study’s validity. For example, missing data during the night may not be a problem for a study interested in identifying daytime behaviors from location data. In our study, we calculated daily average exposure to the weather based on the 24-hourly weather reports from participants’ location [3]. For days with missing data, we imputed participant location. As UK weather stations are approximately 40 km apart, missing information on small relocations would not result in assigning participants to the wrong weather station. Furthermore, misclassification would only occur if the weather conditions at the “wrong” weather station were sufficiently different to change a participant’s daily average exposure. Most previous studies investigating weather and pain measured participants’ location only once and used daily weather reports, rather than hourly [26]. Compared to those studies, weather exposure in our study is less likely to be misclassified, even for participants with only 1 or 2 observed locations per day.

Participant age and sex did not predict missing location data, suggesting that data completeness is not associated with those 2 demographic factors. However, the difference in location-data completeness between iPhone and Android users could be a source of bias. Just-in-time interventions that depend on location data could be less safe and effective for iPhone users compared to Android users. On average, Android users have a lower socioeconomic status than iPhone users—a factor that is related to many health outcomes and may be associated with health disparities in underprivileged groups [27-29]. In observational studies, this difference could introduce selection bias. For example, exclusion of participants with incomplete data (complete case analysis) could lead to results that do not generalize to wealthier iPhone users.

Observational studies could impute missing location data based on participants’ past behavior [30,31]. In that case, it is important to assess whether the imputation algorithm is also valid for iPhone users who may have fewer past data points available. If imputation is not feasible, researchers may want to consider using different devices to collect location data, such as a GPS tracker, which may be more suitable to answer certain research questions requiring complete location data for short periods of time [4,9]. Of note, although the imputation would mitigate some threats to internal validity due to selection bias, they do not address external validity. Study results may still not be generalizable to the wider population, especially not to underserved communities that tend to use health technologies less and may have fewer financial resources to purchase smartphones and pay for connection maintenance [29].

### Improving Location-Data Completeness

At study design, researchers should optimize app settings and user instructions to improve location-data completeness. Our study showed that location was more often recorded around survey completion and around push notifications. Thus, encouraging participants to complete surveys and sending push notifications may improve location-data completeness as well as survey responses. As Android phone users have higher location-data completeness than iPhone users, restricting participation to Android users could improve location-data completeness. However, it could introduce important limitations to generalizability, given that many people have iPhones (market share 27% worldwide [32] and 54% in the United States [33]).

### Conclusion

Missing hourly smartphone location data is common: in our study, 63% of hourly data points were missing. Missing data were more likely for iPhone users, during the night, on weekend days, and if participants had not recently used the app to complete a survey. Participant age and sex did not predict missing location data. Differences in location-data completeness between iPhone and Android users may impact the validity of observational or interventional studies. The predictors of missing data can help researchers at study design to optimize app settings and user instructions for higher location-data completeness. In addition, it may inform their assessment of context-specific consequences of missing location data.

## Abbreviations

OR: odds ratio

## References

[1] Amor J, James C (2015). Setting the scene: Mobile and wearable technology for managing healthcare and wellbeing.

[2] Schultz DM, Beukenhorst AL, Yimer BB, Cook L, Pisaniello H, House T, Gamble C, Sergeant JC, McBeth J, Dixon WG (2020). Weather Patterns Associated With Pain In Chronic-Pain Sufferers. Bull Am Meteorol Soc 2020.

[3] Dixon WG, Beukenhorst AL, Yimer BB, Cook L, Gasparrini A, El-Hay T, Hellman B, James B, Vicedo-Cabrera AM, Maclure M, Silva R, Ainsworth J, Pisaniello HL, House T, Lunt M, Gamble C, Sanders C, Schultz DM, Sergeant JC, McBeth J (2019). How the weather affects the pain of citizen scientists using a smartphone app. NPJ Digit Med.

[4] Glasgow ML, Rudra CB, Yoo E, Demirbas M, Merriman J, Nayak P, Crabtree-Ide C, Szpiro AA, Rudra A, Wactawski-Wende J, Mu L (2016). Using smartphones to collect time-activity data for long-term personal-level air pollution exposure assessment. J Expo Sci Environ Epidemiol.

[5] Lee EW, Bekalu MA, McCloud R, Vallone D, Arya M, Osgood N, Li X, Minsky S, Viswanath K (2020). The Potential of Smartphone Apps in Informing Protobacco and Antitobacco Messaging Efforts Among Underserved Communities: Longitudinal Observational Study. J Med Internet Res.

[6] Marsh A, Hirve S, Lele P, Chavan U, Bhattacharjee T, Nair H, Campbell H, Juvekar S (2020). Validating a GPS-based approach to detect health facility visits against maternal response to prompted recall survey. J Glob Health.

[7] Dale LP, White L, Mitchell M, Faulkner G (2019). Smartphone app uses loyalty point incentives and push notifications to encourage influenza vaccine uptake. Vaccine.

[8] Donaire-Gonzalez D, Valentín Antònia, de Nazelle A, Ambros A, Carrasco-Turigas G, Seto E, Jerrett M, Nieuwenhuijsen MJ (2016). Benefits of Mobile Phone Technology for Personal Environmental Monitoring. JMIR Mhealth Uhealth.

[9] Goodspeed R, Yan X, Hardy J, Vydiswaran VV, Berrocal VJ, Clarke P, Romero DM, Gomez-Lopez IN, Veinot T (2018). Comparing the Data Quality of Global Positioning System Devices and Mobile Phones for Assessing Relationships Between Place, Mobility, and Health: Field Study. JMIR Mhealth Uhealth.

[10] Boonstra TW, Nicholas J, Wong QJ, Shaw F, Townsend S, Christensen H (2018). Using Mobile Phone Sensor Technology for Mental Health Research: Integrated Analysis to Identify Hidden Challenges and Potential Solutions. J Med Internet Res.

[11] Vhaduri S, Poellabauer C, Striegel A, Lizardo O, Hachen D (2017). Discovering places of interest using sensor data from smartphones and wearables. IEEE Xplore.

[12] Krenn PJ, Titze S, Oja P, Jones A, Ogilvie D (2011). Use of global positioning systems to study physical activity and the environment: a systematic review. Am J Prev Med.

[13] Marsh A, Hirve S, Lele P, Chavan U, Bhattacharjee T, Nair H, Campbell H, Juvekar S (2020). Validating a GPS-based approach to detect health facility visits against maternal response to prompted recall survey. J Glob Health.

[14] Do TMT, Dousse O, Miettinen M, Gatica-Perez D (2015). A probabilistic kernel method for human mobility prediction with smartphones. Pervasive and Mobile Computing.

[15] Torous J, Staples P, Barnett I, Sandoval LR, Keshavan M, Onnela JP (2018). Characterizing the clinical relevance of digital phenotyping data quality with applications to a cohort with schizophrenia. NPJ Digit Med.

[16] Gelman A (2005). Analysis of variance—why it is more important than ever. Ann. Statist.

[17] Vittinghoff E, Glidden D, Shiboski SM (2012). Repeated measures and longitudinal data analysis. Regression Methods in Biostatistics.

[18] Bates D, Maechler M, Bolker B, Walker S, Christensen RHB, Singmann H, Dai B, Scheipl F, Grothendieck G (2021). Package ‘lme4’. Linear mixed-effects models using S4 classes. R Package Version 2021.

[19] Gelman A, Hill J (2006). Data Analysis Using Regression and Multilevel/Hierarchical Models.

[20] Knowles J, Frederick C merTools: tools for analyzing mixed effect regression models. R Package version 050 2019.

[21] Bähr S, Haas G, Keusch F, Kreuter F, Trappmann M (2020). Missing Data and Other Measurement Quality Issues in Mobile Geolocation Sensor Data. Social Science Computer Review.

[22] Kiang MV, Chen J, Krieger N, Buckee C, Alexander M, Baker J, Buckner R, Coombs G, Rich-Edwards J, Carlson K, Onnela JP (2021). Sociodemographic Characteristics of Missing Data in Digital Phenotyping. Scientific Reports.

[23] Beukenhorst AL, Schultz DM, McBeth J, Lakshminarayana R, Sergeant JC, Dixon WG (2017). Using Smartphones for Research Outside Clinical Settings: How Operating Systems, App Developers, and Users Determine Geolocation Data Quality in mHealth Studies. Stud Health Technol Inform.

[24] Torous J, Kiang MV, Lorme J, Onnela JP (2016). New Tools for New Research in Psychiatry: A Scalable and Customizable Platform to Empower Data Driven Smartphone Research. JMIR Ment Health.

[25] Torous J, Staples P, Barnett I, Sandoval LR, Keshavan M, Onnela JP (2018). Characterizing the clinical relevance of digital phenotyping data quality with applications to a cohort with schizophrenia. NPJ Digit Med.

[26] Beukenhorst AL, Schultz DM, McBeth J, Sergeant J, Dixon W (2020). Are weather conditions associated with chronic musculoskeletal pain? Review of results and methodologies. Pain.

[27] Dorsey ER, Chan YF, McConnell MV, Shaw SY, Trister AD, Friend SH (2017). The Use of Smartphones for Health Research. Acad Med.

[28] Onnela JP (2021). Opportunities and challenges in the collection and analysis of digital phenotyping data. Neuropsychopharmacology.

[29] Lee E, Viswanath K (2020). Big Data in Context: Addressing the Twin Perils of Data Absenteeism and Chauvinism in the Context of Health Disparities Research. J Med Internet Res.

[30] Barnett I, Onnela JP (2020). Inferring mobility measures from GPS traces with missing data. Biostatistics.

[31] Song C, Qu Z, Blumm N, Barabási AL (2010). Limits of predictability in human mobility. Science.

[32] O'Dea S (2012). Mobile operating systems: market share worldwide from January 2012 to October 2021. Statista.

[33] O'Dea S Market share of mobile operating systems in North America from January 2018 to June 2021. Statista.

